# Extensive Direct Subcortical Cerebellum-Basal Ganglia Connections in Human Brain as Revealed by Constrained Spherical Deconvolution Tractography

**DOI:** 10.3389/fnana.2016.00029

**Published:** 2016-03-18

**Authors:** Demetrio Milardi, Alessandro Arrigo, Giuseppe Anastasi, Alberto Cacciola, Silvia Marino, Enricomaria Mormina, Alessandro Calamuneri, Daniele Bruschetta, Giuseppina Cutroneo, Fabio Trimarchi, Angelo Quartarone

**Affiliations:** ^1^IRCCS Centro Neurolesi “Bonino Pulejo”, MessinaItaly; ^2^Department of Biomedical Sciences and of Morphological and Functional Images, University of MessinaMessina, Italy

**Keywords:** basal ganglia, cerebellum, connectivity, CSD, tractography

## Abstract

The connections between the cerebellum and basal ganglia were assumed to occur at the level of neocortex. However evidences from animal data have challenged this old perspective showing extensive subcortical pathways linking the cerebellum with the basal ganglia. Here we tested the hypothesis if these connections also exist between the cerebellum and basal ganglia in the human brain by using diffusion magnetic resonance imaging and tractography. Fifteen healthy subjects were analyzed by using constrained spherical deconvolution technique obtained with a 3T magnetic resonance imaging scanner. We found extensive connections running between the subthalamic nucleus and cerebellar cortex and, as novel result, we demonstrated a direct route linking the dentate nucleus to the internal globus pallidus as well as to the substantia nigra. These findings may open a new scenario on the interpretation of basal ganglia disorders.

## Introduction

Traditionally, the basal ganglia are thought to play a role in the selection and inhibition of motor commands, while the cerebellum plays a role in tuning and reshaping ongoing movement. Though the functions of these regions are often addressed separately for heuristic reasons, normal motor behavior requires seamless integration.

The connections between cerebellum and cerebral cortex have been considered in the past to be anatomically and functionally distinct from those linking basal ganglia with cerebral cortex (Doya, 2000; Graybiel, 2005).

Any interactions between cerebro-cerebellar and cerebro-basal ganglia circuits were assumed to occur primarily at the level of neocortex. However evidences from recent anatomical experiments, using retrograde trans-neuronal transport of rabies virus, have challenged this old perspective demonstrating di-synaptic subcortical pathways that directly link the cerebellum with the basal ganglia (Bostan et al., 2013).

In rats, the lateral deep cerebellar nucleus was found to project to the dorsolateral striatum via the central lateral nucleus of the thalamus (Ichinohe et al., 2000). Moreover, it was shown in primates, that the dentate and interpositus deep cerebellar nuclei send projections via di-synaptic or tri-synaptic pathways to the putamen or external segment of the globus pallidus (GP), with Th as an intermediate relay station (McFarland and Haber, 2000; Hoshi et al., 2005). In addition, anatomical studies in rats and cats have also suggested the existence of a pathway starting from deep cerebellar nuclei and reaching nigro-striatal dopamine neurons (Snider et al., 1976). Although all these connections were found to exit from cerebellum (efferent projections), also an afferent pathway connecting basal ganglia and cerebellum was described (Bostan et al., 2010).

Despite neuroanatomical tract-tracing methods still represent the gold standard for studying the anatomo-physiology of basal ganglia, the application of these techniques to the human brain remains elusive, due to the invasive nature of such methods (McFarland and Haber, 2000; Nambu et al., 2000; Kita, 2001; Lu, 2011). Therefore, whether these specific connections between the basal ganglia and cerebellum exist in the human brain remains still unclear.

On the other hand, recent developments in diffusion magnetic resonance imaging (dMRI) and tractography allows for non-invasive and *in vivo* investigation of the anatomical substrate of basal ganglia system (Henderson, 2012). It is known that conventional Diffusion Tensor Imaging (DTI) suffers from several limitations making this approach unable to distinguish, within each voxel, fibers with multiple orientations (Tournier et al., 2011).

One promising approach is constrained spherical deconvolution (CSD), a diffusion modeling technique that allows reliable estimation of one or more fiber directions in presence of intravoxel orientational heterogeneity (Tournier et al., 2007, 2008).

In addition this approach overcomes partial volumes effects associated with DTI, allowing a reliable estimation of diffusion parameters (Tournier et al., 2008). Therefore CSD-based tractography, with respect to standard DTI, may increase the sensitivity to detect white matter abnormalities in complex anatomical tracts. It is worthy to note that tractography does not directly visualize the axonal fibers, allowing only a reconstruction of their trajectories inferred based on water local diffusion. Using this innovative approach we have recently shown that it is possible to reconstruct complex white matter networks, such as basal ganglia, claustrum and limbic circuits (Arrigo et al., 2014; Milardi et al., 2015a,b; Mormina et al., 2015). Here, we tested the hypothesis if connections between cerebellum and basal ganglia, already described in animals, might also exist in human brain.

## Materials and Methods

### Participants

The research followed the tenets of the Declaration of Helsinki; written informed consent was signed from all included subjects, after explanation of the nature and possible consequences of the procedure. The study was approved by the institutional review board of IRCCS Bonino Pulejo, Messina, Italy (Scientific Institute for Research, Hospitalization and Health Care).

A total of 15 human subjects (mean age 29; age range 25–32 years) were recruited. No participant had any history of any overt neurological disease.

### Data Acquisition

The study was performed with a 3T Achieva Philips scanner using a 32-channels SENSE head coil. In each patient the following MRI sequences were carried out:

(1) 3D high-resolution T1 weighted Fast Field Echo (FFE) sequence was acquired using the following parameters: repetition time 25 ms; echo time 4.6 ms; flip angle 30°; FOV 240 mm × 240 mm; reconstruction matrix 240 × 240; voxel size 1 mm × 1 mm × 1 mm; slice thickness 1 mm. The acquisition time was 6 min.
(2) 3D high-resolution T2 weighted Turbo Spin Echo (TSE) sequence was obtained using the following parameters: repetition time 2,500 ms; echo time 380 ms; FOV 250 mm × 250 mm; reconstruction matrix 312 × 312; voxel size 0.8 mm × 0.8 mm × 0.8 mm; slice thickness 0.8 mm. The acquisition time was 9 min and 38 s.
(3) A dual phase encoded pulsed gradient spin echo diffusion weighted sequence (Embleton et al., 2010) using 60 gradient diffusion directions chosen following an electrostatic repulsion model (Jones et al., 1999). The other sequence parameters were: diffusion weighting b-factor 1200 s/mm^2^; repetition time 11884 ms; echo time 54 ms; FOV 240 mm × 240 mm; scan matrix 112 × 112; reconstruction matrix 256 × 256; axial slice thickness 2 mm; no inter-slice gap.

### Data Preprocessing

Subject motion within the scanner during acquisition may compromise analysis of diffusion weighted images (DWIs); thus, for each subject, DWIs were corrected for motion as well as for susceptibility distortion artifacts using tools available within SPM8 Matlab toolbox^1^; rotational part of transformations were later applied to individual gradient directions.

For each subject, in order to align with high precision structural scans (T1 and T2) onto preprocessed diffusion images, a coregistration pipeline, outlined by Besson and co-workers was performed (Besson et al., 2014). More in details, cerebral spinal fluid (CSF) component was segmented out from b0 unweighted diffusion image and T1w scans using New Segment option of SPM8. CSF coming from T1w image was then warped to match CSF coming from b0-image; FLIRT and FNIRT FSL utilities were used to this end^2^. Estimated normalization was eventually applied to T1w image.

To coregister T2w scan, a rigid co-registration of the latter to T1w image was carried out. Eventually, the same normalization previously estimated was applied.

By using such non-linear procedure we attempted to minimize possible misalignment biases coming from usage, in diffusion images space, of ROIs segmented in space of structural scans.

### Diffusion Signal Model

To model diffusion signal, we used a modified High Angular Resolution Diffusion Imaging (HARDI) technique called CSD: this technique estimates, for each voxel, a fiber Orientation Distribution Function (fODF). fODF is a continuous function of the sphere which attempts to detect the portion of signal coming from a fiber bundle pointing in a given direction. The main hypothesis underlying such model is that characteristic fiber bundle signal is known and that can be approximated by the so-called single fiber response function (Tournier et al., 2007). Such response function was estimated from the data; to obtain a reliable estimate, we performed calculations using only voxels with high likelihood of being crossed by a single fiber bundle. Identification of such voxels was obtained by calculating Fractional Anisotropy index (FA) in all WM voxels (Pierpaoli and Basser, 1996); only voxels showing a FA bigger than 0.7 were taken into account to estimate response function. Such cutoff is indeed known to be a strong indicator of the presence of a unique fiber population insisting over a voxel. Response function calculation and fODFs fitting were obtained using MRtrix2 software package^3^. In our study, spherical harmonic degree was fixed equal to 8 in order to obtain robustness to noise.

Using CSD to extract local fiber orientations we managed to overcome partial volume effects associated with standard DTI and also to improve, the poor angular resolution achieved with QBI (Q-balls Imaging), while discarding DSI due to its longer acquisition time (Tournier et al., 2008). It is known that higher b-values permit to resolve smaller angles among fibers (Alexander and Barker, 2005; Tournier et al., 2007); on the other side they require longer acquisition times, thus increasing the probability to detect motion related artifacts. Thus, we preferred a lower *b*-value in order to obtain a good quality/speed trade-off.

### Tractography

Tractography permits to detect major WM fiber bundles non-invasively (Conturo et al., 1999; Mori et al., 1999). Starting from a voxel of interest (seed voxel) a numerical process allows to follow WM bundles route on the basis of diffusion signal in neighboring voxels. The resulting tract, called streamline, increases its size until some stopping criteria are met. Due to the noisy nature of DW images, a streamline may follow biologically non-meaningful pathways (false positive); moreover, if a single streamline is initialized for each seed voxel (deterministic approach), reconstruction fails in presence of voxels with complex bundles geometries (like crossing, kissing and branching fibers) (Jeurissen et al., 2013). As an alternative, a probabilistic algorithm can be adopted, i.e., a number of streamlines can be run from each voxel (Lazar and Alexander, 2005; Whitcher et al., 2008; Jeurissen et al., 2011): at each reconstruction step a certain direction is chosen, the widespread of whom is based on the uncertainty estimated from the data themselves. In this article, a probabilistic algorithm was adopted.

Of note, if from one side CSD combined with a probabilistic propagation permits to track bundles even crossing voxels with complex geometries, on the other hand the number of false positive tends to increase. Hence, criteria for initializing and stopping a streamline as well as spatial priors have to be carefully defined to guide tractography. In this paper, following conditions were adopted: step size = 0.2 mm, maximum angle = 10°, minimal fODF amplitude = 0.15 (this is a more conservative choice with respect to usual standards) (Descoteaux et al., 2009; Tournier et al., 2011). Moreover, we allowed streamlines to be sampled within a WM mask obtained by segmenting co-registered T1 scans. By using such spatial prior, we avoid tracts to reach unrealistic regions, like deep GM or CSF.

We performed, for each subject, probabilistic whole brain tractography by generating ten millions streamlines using WM masks previously estimated both as seed and mask ROIs; before running tractography, we applied a small dilatation to WM masks in order to allow streamlines to reach our ROIs, placed in GM, for subsequent analyses.

For visualization purposes, we reconstructed a color-coded map in which red, blue, and green colors indicate the principal streamline directions (Pajevic and Pierpaoli, 1999). Specifically, red color indicates a left–right pattern, green color an anterior–posterior pattern, and blue color a caudal–cranial pattern. Intensity and pureness of these colors vary according to the behavior of fiber bundles in all intermediate positions.

### Segmentation

**FIGURE 1:**
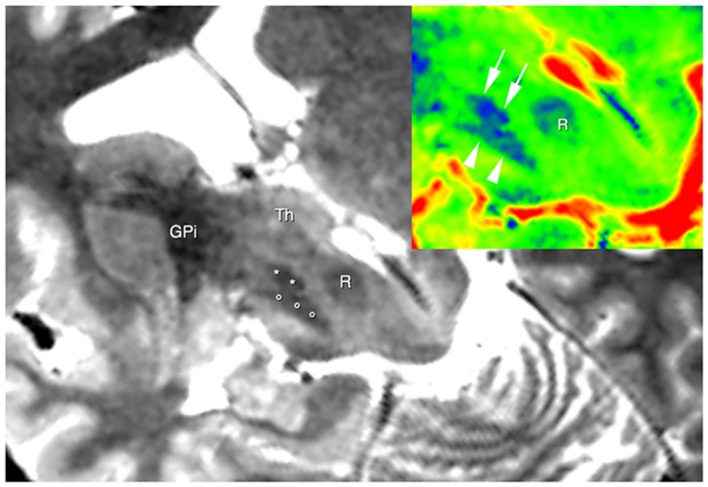
**Brain areas segmentation.** A semi-oblique axial view of 3D T2 weighted images shows the right substantia nigra (circles), the right subthalamic nucleus (STN; asterisks), and the right internal globus pallidus GPI. Thalamus (Th) can be also seen. In the top right corner, the same slice is shown; now a different colormap (jet) was used to display it. Here; advantages of using T2w image are clear, as evident from the substantia nigra (arrowheads), the STN (arrows) and the red nucleus (R).

The use of 3D TSE sequence permitted to obtain high-resolution images with a relative short acquisition time. At same time this sequence allowed to obtain a fine representation of the iron loaded nuclei due to T2* effect linked with the use of a very long echo-time (**Figure 1**).

Segmentation was carried out manually by a skilled radiologist based on previously co-registered structural T1 and T2 images by means of a viewer provided together with MRtrix package.

#### Substantia Nigra (SN)

The mask for Substantia Nigra (SN) was outlined bilaterally in T2-weighted images in the axial plane. We localized the SN immediately above the pons, as a hypointense region between crus cerebri and tegmentum. Ascending in caudo-cranial direction, this region appeared to be expanded ventrally and we localized behind it the red nucleus (RN), in a more medial position. We were able to identify VTA as the medial hyperintense zone between RN and SN. In the upper sections of SN, we identified the subthalamic nucleus (STN) as dorso-lateral boundary and marked the SN in every ascending slices until the superior colliculi and the cerebral aqueduct disappeared. As posterior and anterior boundaries of the most superior section, the cerebral aqueduct and mammillary bodies were, respectively, delineated. STN becomes prominent in the axial plane including third ventricle, RN and the posterior commissure. The mask was better defined in both sagittal and coronal planes.

#### Red Nucleus

The mask for RN was outlined bilaterally in T2-weighted images in the axial plane. We localized the RN in a medial position in ascending sections of the midbrain, at the level of the superior colliculi, behind the SN, with VTA as ventro-medial boundary. Its almost cylindrical shape results in an approximately circular hypointense region in transverse section. In the upper sections, STN appears to be located in a ventro-lateral position to RN. The position of the mask was confirmed in both sagittal and coronal planes.

#### STN of Luys

The mask for STN was outlined bilaterally in T2 weighted images in coronal plane. Proceeding in dorso-ventral direction, the RN was first localized, and when it disappeared the STN appeared as a hypointense lens-shaped region, with its major axis tilted upward by almost 45°. The inferior and ventro-medial boundaries for STN were individuated by SN, whilst the zona incerta marked the dorso-lateral boundary. H1 and H2 Forel’s fields and the posterior limb of the internal capsule identified, respectively, the dorso-medial and lateral boundaries. STN was outlined in dorso-ventral direction until SN completely disappeared. The position of the mask was confirmed in both sagittal and axial planes.

#### Thalamus

The mask for Th was outlined in T1-weighted images on the coronal plane. The outlining of Th was performed in a ventro-dorsal direction. In the anterior sections, stria terminalis was used as medial boundary until third ventricle and adhaesio intertalamica appeared; internal capsule was used as lateral and ventro-lateral boundary, while the hypothalamic area represented the inferior and ventro-medial limit. As dorsal boundary, we identified the lateral ventricle dorso-medially and the caudate nucleus and the genu of the internal capsule dorso-laterally. The mask was then better defined in sagittal and axial planes.

#### Putamen

The putamen mask was outlined in the axial plane in T1-weighted images. The external capsule was chosen as the lateral border; the anterior limb of the internal capsule was chosen as the ventro-medial boundary while the posterior limb of the internal capsule as the postero-medial boundary. The position of the mask was confirmed in both sagittal and coronal planes.

#### Caudate Nucleus

Caudate nucleus was outlined in T1-weighted images in axial plane. The lateral ventricle gave the medial boundary, while the anterior limb of internal capsule was chosen as the lateral limit. The head of the caudate nucleus was bordered ventrally by the nucleus accumbens septi and dorsally by the stria terminalis and subcallosal stratus. The accuracy of mask positioning was controlled in sagittal and coronal planes.

#### Globus Pallidus

The mask of the GP was outlined in the coronal plane in T2-weighted images. It appeared with a hypointense triangular shape that progressively disappeared in ventro-dorsal direction. The anterior limb of the internal capsule was used as a dorso-medial boundary, while genu and posterior limb were used as a medial border. Lamina medullaris was used as lateral border between GP and putamen. The mask positioning was controlled in sagittal and axial plane.

### Connectivity Analysis

Once whole brain probabilistic tractography process terminated, for each subject we isolated streamlines linking ROIs previously segmented on structural scans. In this way, for each couple or ROI, we were able to calculate number of streamlines connecting them; with some limitations (Smith et al., 2013), such numbers are used in specialized literature as markers of connectivity density, either for analyses on healthy brains or in pathological contexts (Behrens and Sporns, 2012; Bijttebier et al., 2015; Li et al., 2016). Those measures were obtained by means of Mrtrix2 package; analyses were performed by means of in-house scripts built with MATLAB Software Package^4^, release 2013b.

## Results

**FIGURE 2:**
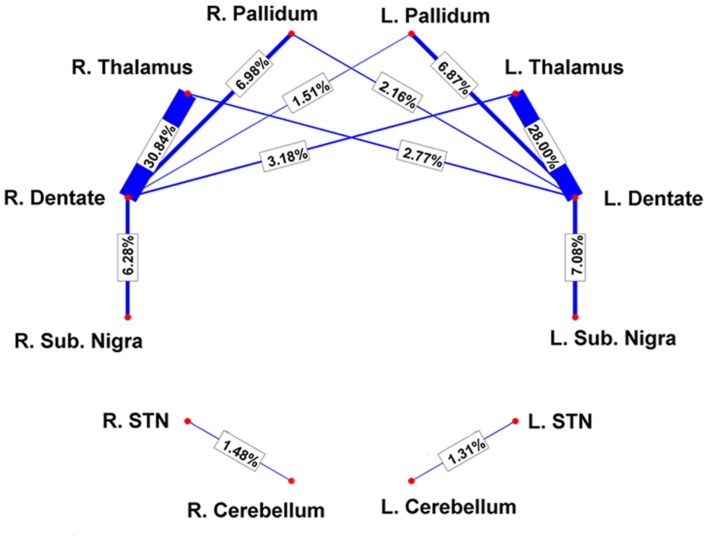
**Connectivity results are visualized by means of a graph.** Red nodes correspond to specific brain areas (labeled near to them). Edges (in blue) correspond to the pathways analyzed. Edge thickness is proportional to connectivity strength as measured from individual subjects; average weights are reported on top of each corresponding edge as well.

By using a fine segmentation of the SN, GP, STN, dentate, and cerebellar cortex bilaterally (**Figure 1**), we detected, in all subjects, connections between cerebellum and basal ganglia system. **Table 1** reports connectivity results obtained after averaging the individual connectivity profiles, while **Figure 2** shows them by means of an undirected graph. Analysis of coefficient of variation (COV) observed for each connectivity showed differential results for reconstructed pathways. Ipsilateral connections between thalami and dentate nuclei were quite consistent (see **Table 1**); similar considerations can be made for ipsilateral left pathway connecting STN and Cerebellum (see **Table 1**). A good consistence between subjects was even observed for contralateral connections Th and dentate nuclei and for ipsilateral connections between dentate nuclei and pallidal nuclei, with COVs ranging between 0.10 and 0.20, Bigger COVs were observed for other connections, thus showing an higher variability in the density strengths.

**Table 1:** Connectivity profile of anatomical connections investigated, averaged from individual subject profiles.

Target A	Target B	Laterality	Mean (%)	*SD* (%)	COV
R. Dentate	R. Thalamus	Ipsilateral	30.84	5.73	0.19
L. Dentate	L. Thalamus	Ipsilateral	28	2.45	0.09
L. Dentate	L. Substantia Nigra	Ipsilateral	7.08	3.51	0.50
R. Dentate	R. Pallidum	Ipsilateral	6.978	1.25	0.18
L. Dentate	L. Pallidum	Ipsilateral	6.87	0.77	0.11
R. Dentate	R. Substantia Nigra	Ipsilateral	6.28	2.82	0.45
R. STN	R. Cerebellum	Ipsilateral	1.48	1.04	0.80
L. STN	L. Cerebellum	Ipsilateral	1.31	0.1	0.08
R. Dentate	L. Thalamus	Contralateral	3.18	0.39	0.12
L. Dentate	R. Thalamus	Contralateral	2.77	0.44	0.16
L. Dentate	R. Pallidum	Contralateral	2.16	0.97	0.45
R. Dentate	L. Pallidum	Contralateral	1.51	0.54	0.36

*Standard deviation con Coefficient of Variation (STD and COV, respectively) are shown to highlight inter-subject variability observed for each reconstructed pathway.*

### Dento-Thalamic Pathways

Since dento-thalamic pathways are well documented in animal and human anatomical literature we decide to include these tracts in the connectivity analysis to give an idea of the overall strength of cerebello-basal ganglia connections in comparison to this well-known pathway (see **Table 1**).

**FIGURE 3:**
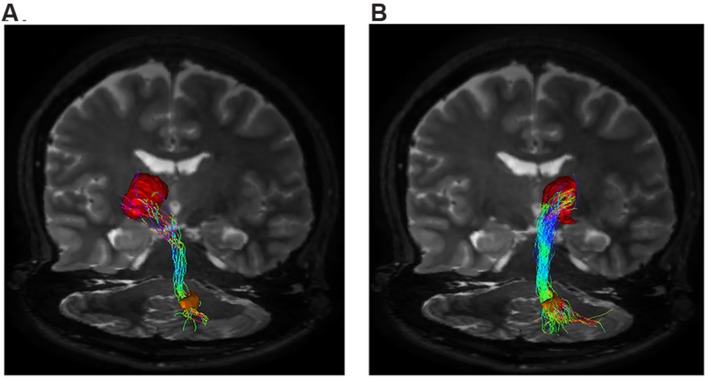
**Dento-thalamic pathways.** Representation of the dento-thalamo connections. Dentate nucleus (orange VOI) and thalamus (red VOI) are connected by a direct pathway. Connections between thalamus and striatum complete the circuit. **(A)** Coronal view of the pathway connecting the right dentate nucleus (orange VOI) to the left thalamus (red VOI). The fibers exited the cerebellum via the right superior cerebellar peduncle, crossed the midline at the level of the midbrain (decussation of brachium conjunctivum) and reached the contralateral thalamus through the left cerebral peduncle. **(B)** Coronal view of the pathway connecting the right dentate nucleus (orange VOI) to the ipsilateral thalamus (red VOI). The fibers exited the cerebellum via the right superior cerebellar peduncle, ran through the midbrain and reached the thalamus through the right cerebral peduncle.

Our tractographic findings showed dento-thalamic streamlines connecting dentate nucleus to the contralateral Th. This bundle was found to run through the superior cerebellar peduncle, decussating at the level of midbrain, in the decussation of brachium conjunctivum (**Figure 3**). Our connectivity analysis showed also a strong ipsilateral component (see **Table 1**), passing through the superior cerebellar peduncle and reaching the ipsilateral Th.

### STN-Cerebellar Cortex Connections

**FIGURE 4:**
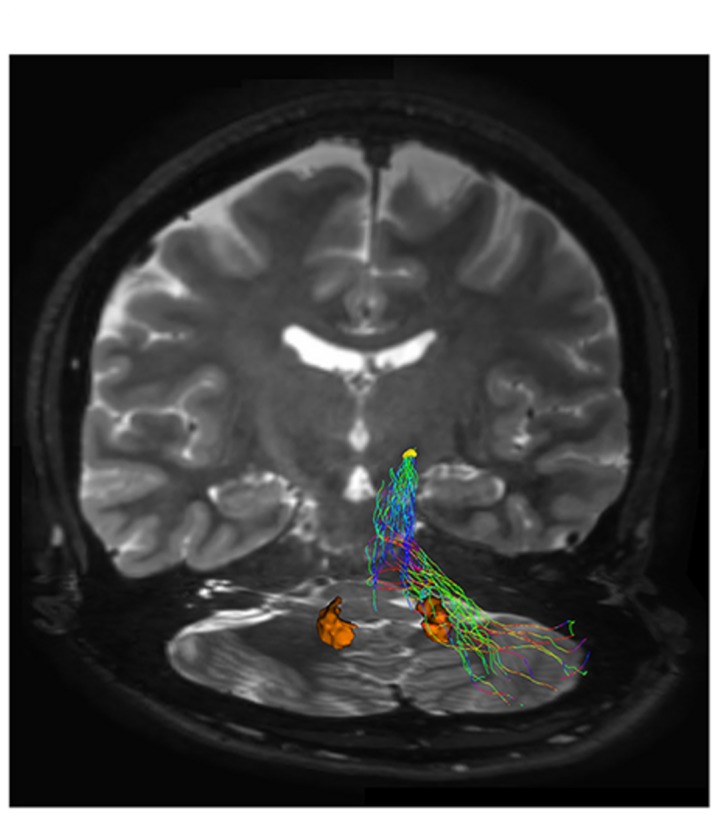
**Subthalamic-cerebellar cortex connections.** The ipsilateral cerebello-subthalamic connection linking cerebellar cortex with ipsilateral STN (yellow VOI) is showed on frontal view. Please note that this pathway also run externally to dentate nucleus (orange VOI).

We reconstructed a pathway connecting cerebellar cortex to STN (yellow VOI). The connectivity analysis is reported in **Table 1**. The anatomical course of this bundle, based on our findings, includes its passage through the middle cerebellar peduncle and the ventral-basilar area of the pons, as shown in **Figure 4**.

### Dentate-Nigral Connections

**FIGURE 5:**
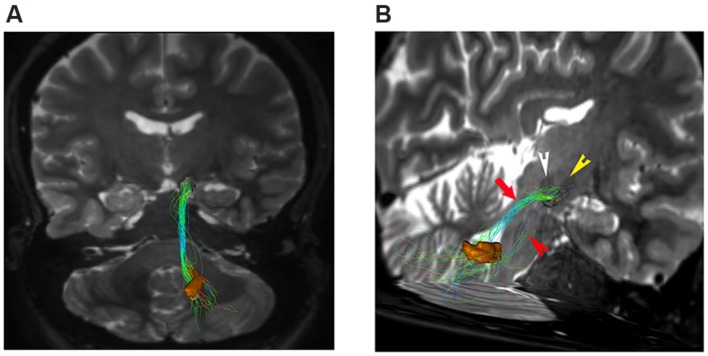
**Dentate-nigral connections. (A)** Coronal view shows the connections between the right dentate nucleus (orange VOI) and the ipsilateral substantia nigra (black VOI). **(B)** An oblique para-coronal view allows evaluating the white matter bundle connecting the right dentate nucleus (orange VOI) to the ipsilateral subtantia nigra (black VOI). The bulk of this white matter bundle passes through the superior cerebellar peduncle (red arrow) with a small component running through the middle cerebellar peduncle (red arrowhead). Note the hypointense iron-loaded RN (white arrowhead) and STN (yellow arrowhead) close to the substantia nigra.

In our analysis, strong connections are represented by dentate-nigral pathway (**Figure 5**).

**FIGURE 6:**
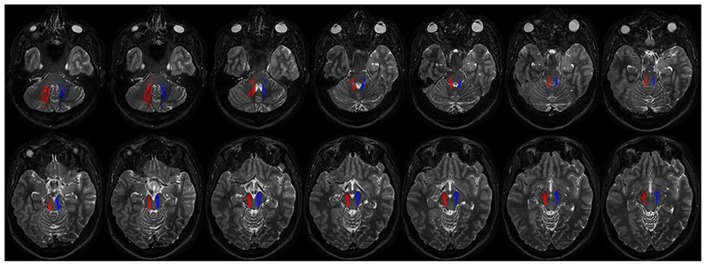
**Course of ipsilateral dentate-nigral connections.** Fourtheen axial T2 weighted scans show the course of the connections between dentate nucleus and substantia nigra. On the right fibers are red colored, on the left side blue colored. The first two images depict tracts leaving the dentate nucleus. From the dentate nucleus the fibers enter the brainstem through the middle and superior cerebellar peduncles and reach the substantia nigra running laterally to the RN.

Connectivity analysis of these fibers is shown in **Table 1**. This bundle was found to run through the superior cerebellar peduncle allowing connection between dentate nucleus and ipsilateral SN (**Figures 5** and **6**).

### Dentate-Pallidal Connections

We found that DN is widely connected with the GP. We identified a strong ipsilateral component and a less represented contralateral one (**Table 1**; **Figure 1**).

**FIGURE 7:**
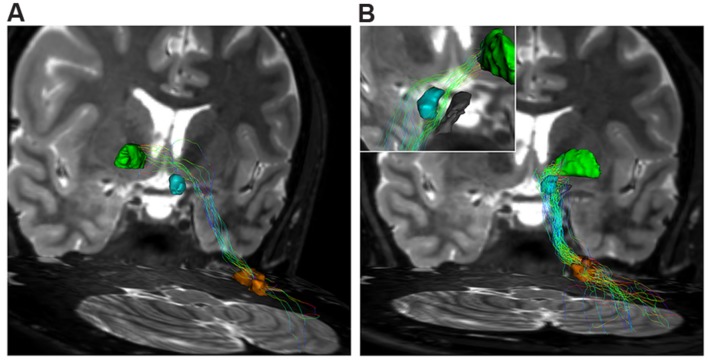
**Dentate-pallidal connections. (A)** Contralateral dento-pallidal connection. Representation on coronal view of the pathway connecting right dentate nucleus (orange VOI) with contralateral globus pallidus (GP; green VOI). The pathway crosses the midline above the RN (cyan VOI) at the level of the midbrain. **(B)** Ipsilateral dento-pallidal connection. Coronal view shows the direct dento-pallidal pathway linking dentate nucleus (orange VOI) to the GP (green VOI). An enlarged view focused on the cranial part of the bundle reveals, with better magnification, the close relationship between the bundle, the RN (cyan VOI) and the substantia nigra (black VOI), which are surrounded by the fibers. Note that the fibers converge mainly on the antero-medial part of the Gpi.

**FIGURE 8:**
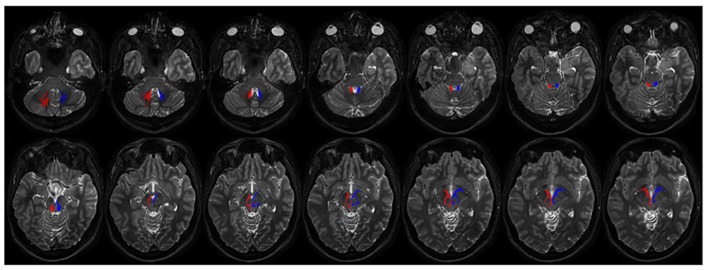
**Course of ipsilateral dentate-pallidal connections.** Representation of the course of the connections between dentate nucleus and GP. On the right fibers are red colored, on the left blue colored. The first two images depict tracts leaving the dentate nuclues. From the dentate nucleus the fibers enter the brainstem through the superior cerebellar peduncle, then they encircle the RN, avoid the substantia nigra and pass through the cerebral peduncle to reach the internal GP.

The contralateral component, beginning from the cerebellum, leaves DN and, after decussating at the level of midbrain above the RN, it reaches the contralateral antero-medial portion of GPi (**Figure 7A**). The ipsilateral component connects the DN, passing through the superior cerebellar peduncle, and reaching the ipsilateral antero-medial portion of GP (**Figures 7B** and **8**).

## Discussion

As a fundamental premise, diffusion-based tractography is a method analyzing the preferential water diffusivity directionality along white matter bundles, thus calculating the highest mathematical probability that water diffuses in a given direction. For this reason, tractography is not sufficient to demonstrate the existence of a specific pathway, if used alone. Further studies with other techniques are needed in order to confirm tractographic findings. In addition, it should be considered that tractographic reconstruction is deeply influenced by the distance between seeding and target regions of interest (ROIs), as well as by their size (Bassett et al., 2001): i.e., the longer the distance a streamline has to cover to reach the final destination, the lower the resulting reconstruction probability. Hence, as a general consideration, the lower values of contralateral connections found in this study, apart from anatomical reasons, could be explained on the basis of the longer distance covered by these pathways, reaching their final targets.

Finally it should be acknowledged that tractography is not able to clearly distinguish monosynaptic connections from multi-synaptic ones as well as the directionality of the signal transmission (afferent or efferent projections).

In the present study we found, for the first time *in vivo* in humans, extensive connections between basal ganglia and cerebellum. In particular, in agreement with previous studies, we provided further support regarding the existence of pathways running between the STN and cerebellar cortex. A novel finding of the present study was the reconstruction of a direct route linking the DN to the GPi and to the substantia nigra.

The functional relevance of this extensive sub-cortical network between basal ganglia and cerebellum is supported by the changes of striatal dopamine release caused, in rats, by cerebellar stimulation or lesions (Neychev et al., 2008). In addition, unilateral electrical stimulation of the cat deep cerebellar nuclei was found to increase dopamine turnover in the contralateral striatum (Nieoullon et al., 1978). On the other hand, unilateral electrolytic lesions of the deep cerebellar nuclei in rats reduce contralateral striatal dopamine turnover (Tellerman et al., 1979).

The STN-ponto-cerebellar pathway, described in the present study, is similar to that described in monkeys by using retrograde virus-tracing technique. In particular, after the injection at the level of neocerebellar cortex, the first order neurons were found in the pontine nuclei while the second order neurons were found in the STN (Bostan et al., 2010). Although we could not provide information regarding transmission direction, we can hypothesize that this pathway is an afferent route, as previously described in animals. This pathway might provide an anatomical basis for understanding how motor and non-motor signals originating from basal ganglia are able to influence cerebellar function, both in healthy and disease states.

Furthermore, the dento-thalamic connections described in the present study may represent the first segment of a larger multisynaptic circuit terminating in the striatum. This is in agreement with Hoshi and associates who demonstrated, by injecting rabies retro-virus in the external part of GPe, that the first-order neurons were located in the striatum, the second-order neurons in the Th and the third-order neurons in deep cerebellar nuclei (Hoshi et al., 2005).

Finally, our findings are in line with other tractographic studies showing the presence of dento-thalamo-striato-pallidal and subthalamo-cerebellar connections in humans (Pelzer et al., 2013).

### Dento-Nigral Pathway

This is the first possible demonstration showing the presence of direct connections between SN and DN. Based upon our segmentation we cannot infer which part of the SN is involved, even if anatomical data would strongly suggest that dentate projecting fibers would probably reach the reticulate SN (SNr) which represents the basal ganglia output. The few evidences available suggest that SNr is not directly related to movement of the limbs but preferentially involved in eye-movement control and non-motor aspects of behaviour (Wichmann and Kliem, 2004). This is perhaps an oversimplification, as Dybdal and associates (Dybdal et al., 2013) have shown that GABA-A agonist muscimol injected into the SNr, induced chorea of the contralateral arm and or leg, contralaterally directed torticollis and contraversive rotational activity. Here the presence of robust connections, between the neocerebellum and the SN, suggest a new complexity of basal ganglia pathophysiology for movement disorders, where SN-DN connections would have a greater role than so far envisaged.

### Dento-Pallidal Connections

Finally, we demonstrated direct connections running between dentate nucleus and the internal portion of GP. Although our results do not represent a proof of evidence for cerebello-pallidal connectivity, they do indicate a good mathematic probability of the existence of this pathway. On the other hand it is worthy to note that a functional connectivity between cerebellum and GPi has been reported in dystonic patients with DBS on GPi, as indexed by a robust alpha band of coherence between these 2 sub-cortical structures, as revealed in a MEG-GP local field potential study. Interestingly, the directionality analysis revealed a flow toward pallidum (Neumann et al., 2015). These direct connections between DN and GPi are very intriguing in light of our recent findings showing the existence of a direct cortico-pallidal pathway (Milardi et al., 2015a).

This cortico-pallidal system is separate from the descending cortico-spinal and cortico-pontine axons, that travel through the internal capsule and bypasses the traditional direct, indirect, and hyperdirect pathways (Milardi et al., 2015a; Smith and Wichmann, 2015). Despite we cannot assume that cortico-pallidal connections and dento–pallidal connections may share the same sub-set of neurons within GPi, it is tempting to speculate that the two systems may somehow interact each other bypassing the striatum. This could open an entirely new perspective into the pathophysiology of basal ganglia and cerebellum.

### Relevance on Motor Control in Normal and Pathological State

Several evidences suggest that cerebellum and basal ganglia are strongly interconnected each other either in physiological and pathological conditions.

Tranchant and colleagues described a woman with torticollis in association with a hemangioma within the cerebellum where FDG-PET revealed marked hypometabolism of the basal ganglia, suggesting that a focal lesion in the cerebellum can produce remote effects within the basal ganglia circuits (Tranchant et al., 1991).

The existence of this consistent cerebellum-basal ganglia sub-cortical network is also supported by significant co-activations of both cerebellum and basal ganglia found in imaging studies that were explicitly designed to study the normal functions of the basal ganglia. For instance, robust cerebellar activation along with activation in the dorsal and ventral striatum has been revealed in experimental models of reward-related learning which are believed to preferentially activate basal ganglia (Doya, 2000; O’Doherty et al., 2003).

Indeed the involvement of cerebellum and basal ganglia in reward-related learning may account why also cerebellum is implied in addiction (Miquel et al., 2009).

Cerebellar interactions with the basal ganglia have been shown to contribute to the symptoms of certain motor disorders, particularly Parkinson’s disease and dystonia (Quartarone and Hallett, 2013; Wu and Hallett, 2013).

For instance, both in parkinsonian patients (Ohye et al., 1974) and in monkey models of the disease (Guehl et al., 2003) an oscillatory activity at tremor frequency has been recorded in regions of the Th receiving cerebellar but not basal ganglia efferents. This is the reason why ventralis intermedious nucleus (VIM) is one of the most effective surgical sites for treating parkinsonian tremor (Narabayashi et al., 1987).

Similarly dystonia can also arise from cerebellar dysfunction and actually may be better described as a network disorder involving basal ganglia and cerebellum (Quartarone and Hallett, 2013; Prudente et al., 2014).

In normal mice with pharmacological excitation of the cerebellum or mutant tottering mice, abnormal cerebellar activity was found to drive dystonic movements (Campbell and Hess, 1998).

Human carriers of genetic mutations associated with dystonia exhibit abnormalities in both the basal ganglia and the cerebellum (Carbon et al., 2008).

One influential line of thought postulates that cerebellum has a compensatory role in dystonia. This is suggested by the correlation between the integrity of cerebello-thalamic tracts and the occurrence of dystonic symptoms (Argyelan et al., 2009), which is also associated with increased motor cortical activation at rest and during movement (Carbon et al., 2011).

Similarly, increased cerebello-motor cortical functional connectivity has been described as a potential compensatory mechanism in patients with writer’s cramp using fMRI that was progressively reduced with larger symptom severity, indicating symptom-related neuroplastic compensatory network changes in dystonia (Dresel et al., 2014).

Finally, it has been recently reported that stimulation of cerebellar cortex by using continuous theta burst transcranial magnetic stimulation may improve dystonia or levodopa-induced dyskinesia in Parkinson’s disease (Koch et al., 2009).

It might be speculated that cerebellar stimulation may modulate pallidal oscillatory activity via an activation of the direct sub-cortical network described in the present study.

In conclusion, the findings of the present study would meet the vision that the cerebello-basal ganglia circuits might consist of several, parallel, segregated, and functionally distinct, but homologous sub-cortical loops that reciprocally connect cerebellum with basal ganglia output relays.

Future studies, using postmortem microsurgery dissection or anterograde and retrograde tracer injection in animals or *in vivo* functional MRI in humans, should be conducted in order to confirm the existence of these new postulated pathways and to clarify their functions.

## Author Contributions

DM: study concepts/study design, data acquisition, data analysis, data interpretation, literature research. AA: study concepts/study design, data analysis, data interpretation. GA: Guarantor of integrity of entire study, approval of final version of submitted manuscript, data interpretation. AC: study concepts/study design, data acquisition, data analysis, data interpretation, literature research. EM: data analysis, data interpretation. AC: statistical analysis. SM: data acquisition, data analysis. DB: literature research. GC: literature research. FT: literature research, manuscript revision. AQ: study concepts/study design, guarantor of integrity of entire study, manuscript revision for important intellectual content.

## Conflict of Interest Statement

The authors declare that the research was conducted in the absence of any commercial or financial relationships that could be construed as a potential conflict of interest.

## Footnotes

1 http://www.fil.ion.ucl.ac.uk/spm/software/spm8/

2 http://fsl.fmrib.ox.ac.uk/fsl/fslwiki/

3 http://jdtournier.github.io/mrtrix-0.2/index.html

4 www.mathworks.com
